# Retroperitoneal or transperitoneal approach in robot‐assisted partial nephrectomy, which one is better?

**DOI:** 10.1002/cam4.3888

**Published:** 2021-05-01

**Authors:** Jing Zhou, Zheng‐Huan Liu, De‐Hong Cao, Zhu‐Feng Peng, Pan Song, Luchen Yang, Liang‐Ren Liu, Qiang Wei, Qiang Dong

**Affiliations:** ^1^ Department of Urology/Institute of Urology West China Hospital Sichuan University Chengdu China; ^2^ Sichuan University Chengdu China; ^3^ Department of Urology West China Hospital Sichuan University Chengdu China

**Keywords:** partial nephrectomy, retroperitoneal, robotic surgical procedures, transperitoneal

## Abstract

**Purpose:**

To systematically assess the perioperative outcomes of retroperitoneal (RP) and transperitoneal (TP) approaches in robot‐assisted partial nephrectomy (RAPN), we conducted an updated meta‐analysis.

**Methods:**

A literature retrieval of multi‐database including PubMed, Web of Science, Embase, Cochrane Library, and CNKI was performed to identify eligible comparative studies from the inception dates to January 2021. Perioperative outcomes included operative time (OT), estimated blood loss (EBL), warm ischemia time (WIT), postoperative length of stay (PLOS), positive surgical margin (PSM), and complications (major complications and overall complications). Outcomes of data were pooled and analyzed with Review Manager 5.4.1.

**Results:**

Twenty‐one studies involving a total of 2482 RP and 3423 TP approach RAPN patients met the inclusion criteria. Operating time (OT) (weighted mean difference [WMD] −16.60; 95% confidence interval [CI] −23.08, −10.12; *p* < 0.01) and PLOS (WMD −0.46 days; 95% CI −0.69, −0.23; *p* < 0.01) were shorter in RP‐RAPN. Besides, lower EBL (WMD −21.67; 95% CI −29.74, −13.60; *p* < 0.05) was also found in RP‐RAPN. Meanwhile, no significant differences were found in other outcomes.

**Conclusions:**

RP‐RARN was superior to TP‐RAPN in patients undergoing RAPN in terms of OT, PLOS, and estimated blood loss. Besides these two approaches have no significant differences in PSMs or perioperative complications.

## Graphical abstract

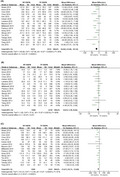

## 1 INTRODUCTION

Due to the increasingly wide application of imaging diagnosis, the detection of renal masses increased dramatically over the past few decades.^1^ There were an estimated new 300,000 cases of renal cell carcinoma (RCC) worldwide in 2016.^2^ Companied with this trend, both theoretical and technical advancement have been made in kidney surgery. Partial nephrectomy (PN) has become an ideal method for most RCC, which is associated with superior perioperative outcomes compared to patients undergoing radical nephrectomy (RN). With the improvement of minimally invasive technology, the prevalence of robot‐assisted PN (RAPN) is growing. Three‐dimensional (3D) magnification view, flexible wristed instruments, and stable cameras make RAPN easier, thus improving perioperative outcomes compared to traditional open PN.^3^

RAPN can be performed with transperitoneal (TP) or retroperitoneal (RP) approach, and each of them has its own advantages and limitations. The increased working space provided by the TP approach allowed adequate manipulating space of the devices, thus decreasing external robotic arm conflict. Conversely, RP approach is performed in the small space of retroperitoneum cavity; therefore, the RP approach tends to be associated with a steeper learning curve.^4^ Nevertheless, there are several potential advantages of the RP approach such as the decreased gastrointestinal morbidity and urine leaks outside of the peritoneum.

Until now, the optimal approach for treating renal masses remains controversial. Therefore, it is necessary to perform a meta‐analysis to systematically compare noteworthy outcomes of two surgical approaches, providing high‐quality medical evidence for the selection of surgical approaches.

## 2 METHODS

### 2.1 Search strategy and inclusion/exclusion criteria

The design of this systematic review was published on the PROSPERO register (https://www.crd.york.ac.uk/PROSPERO/#myprospero ID: CRD42021232640). A literature retrieval of multi‐database including PubMed, Web of Science, Embase, Cochrane Library, and CNKI was performed until January 2021. The search terms were as follows: “robot,” “robotic,” “robot‐assisted,” “robotic‐assisted,” “da Vinci,” and “partial nephrectomy.” No language restriction was used. The reference list of eligible studies and reviewed conference records were also retrieved. Two reviewers independently evaluated all the included studies, and any differences were resolved by consensus.

The inclusion criteria were as follows: (1) a randomized controlled trial or retrospective comparative study or case–control study design; (2) the literature compared RP‐RAPN with TP‐RAPN; (3) studies performed in adults diagnosed with RCC; and (4) including at least one perioperative outcome such as operating time (OT), estimated blood loss (EBL), warm ischemia time (WIT), postoperative length of stay (PLOS), positive surgical margin (PSM), and complication rate. The exclusion criteria were as follows: (1) studies failing to satisfy the inclusion criteria; (2) pediatric patient population; and (3) articles with unavailable results.

### 2.2 Study selection and data extraction

Two reviewers (J. Z. and Z‐H. L.) extracted data from the included studies via reading full‐text articles, respectively. The disagreement was resolved by discussion until consensus was reached. Twenty‐one studies^4^, ^5^, ^6^, ^7^, ^8^, ^9^, ^10^, ^11^, ^12^, ^13^, ^14^, ^15^, ^16^, ^17^, ^18^ published from 2013 to 2021 were included in the meta‐analysis. The following data were pooled from eligible studies: first author, publication date, study type, surgical procedure, number of patients, age, body mass index, tumor size, follow‐up time, and outcome measures (including OT, EBL, WIT, PLOS, overall complications, and major complications). Continuous variables presented as median and interquartile range were converted into mean ± standard deviation according to the methodology described by Hozo et al.^11^

### 2.3 Risk of bias assessments

The Risk of Bias in Non‐Randomized Studies—of Interventions (ROBINS‐I) tool was applied to assess the publication bias by two reviewers independently.^19^ Confounding bias, selection bias, bias in measurement classification of interventions, bias due to deviations from intended interventions, bias due to missing data, bias in measurement of outcomes, and bias in selection of the reported result were included in this tool. Due to the non‐randomized design of all the studies, they were rated high risk of bias for detection and performance.

### 2.4 Quality assessment of studies

The quality of all included studies was evaluated according to the Newcastle–Ottawa Scale (NOS) independently by two reviewers.^20^ Three aspects were included in the assessment of quality: outcome indicators, study group selection, and comparability between groups: 0–2 points for each item, and the full score is 9 points. Studies achieving a score of 7 or more were considered to be of high quality. Besides, the level of evidence for studies included was evaluated based on the criteria published by the Oxford Evidence‐based Medicine Center.^6^

### 2.5 Data analysis

Review Manager Version 5.4.1 (The Cochrane Collaboration) was applied in this study according to the Quality of Reporting of Meta‐analyses (QUOROM) guidelines of the Cochrane Collaboration.^21^ Continuous and dichotomous variables were compared via the weighted mean differences (WMDs) and odds ratios (ORs) with 95% confidence intervals (CIs), respectively. Statistical heterogeneity was assessed using the chi‐squared test, and a *p* value of < 0.05 was considered to indicate statistical significance. The *I* ^2^ statistic was used to appraise the quantity of heterogeneity. The random‐effects (RE) model was used to pool the outcomes of studies with high heterogeneity (*p* < 0.05,*I* ^2^ > 50) among studies. Otherwise, the fixed‐effects (FE) model was used.^22^

## 3 RESULTS

### 3.1 Characteristics of included studies

**FIGURE 1:**
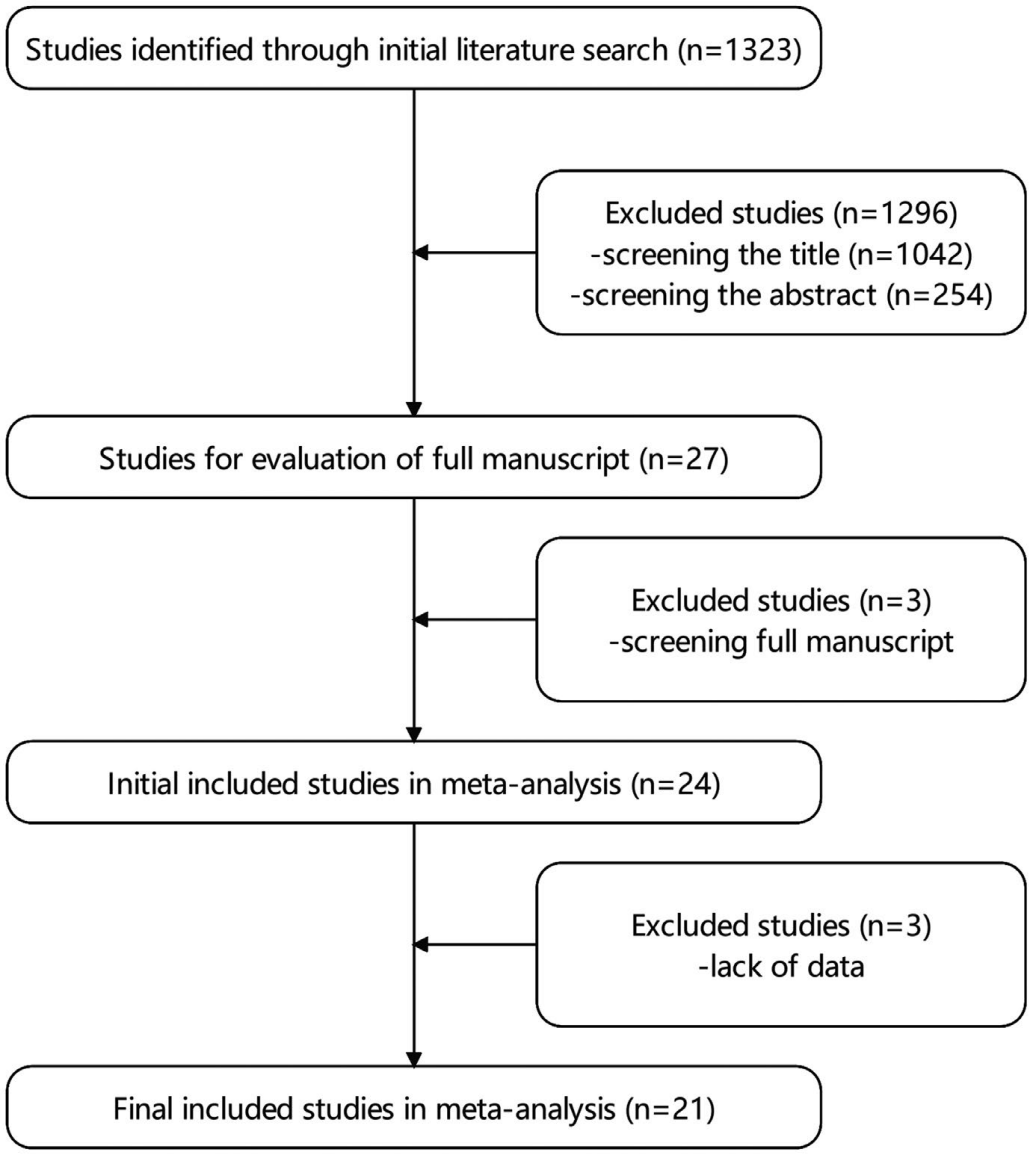
Flowchart diagram of literature search

The process of literature selection is described in the flowchart (Figure 1). Finally, we identified 1323 articles after the initial database search. After screening, 21 studies^4^, ^5^, ^6^, ^7^, ^8^, ^9^, ^10^, ^11^, ^12^, ^13^, ^14^, ^15^, ^16^, ^17^, ^18^ including 5905 patients (TP‐RAPN: 2482 patients; RP‐RAPN: 3423 patients) were eligible for the analysis. All included studies were retrospective studies and were published between 2013 and 2021. The characteristics and the quality evaluation of all the included studies are presented in Tables 1 and 2, respectively, and studies with scores ≥7 were of high quality.

**TABLE 1:** Baseline characteristics of included studies

Author (year)	Design	No. of patients		Age		BMI (kg/m^2^)		Mean tumor size (cm)		Mean R.E.N.A.L. score	
		TP	RP	TP	RP	TP	RP	TP	RP	TP	RP
Hughes‐Hallett et al. (2013)	Retrospective	59	44	60.5	63.3	NA		3.07	2.84	5.5	5.5
Tanaka et al. (2013)	Retrospective	16	10	70	60.5	22.7	23.2	3.4	2.2	7.4	6.1
Gin et al. (2014)	Retrospective	85	55	60	60	NA		NA		8.0	7.0
Choo et al. (2014)	Retrospective	43	43	40.0	53.0	24.5	24.3	2.7	2.8	NA	
Kim et al. (2015)	Retrospective	97	116	58.2	57.2	NA		2.54	2.48	8	8
Tang et al. (2015)	Retrospective	49	33	53.2	52.7	21.6	22.2	2.7	2.8	7.1	6.6
Xia et al. (2016)	Retrospective	44	59	52.1	51.0	NSD		3.8	3.3	NA	
Maurice et al. (2017)	Retrospective	296	74	59	60	29.4	30.0	2.5	2.4	7	7
Stroup et al. (2017)	Retrospective	263	141	58.0	59.3	28.6	29.8	3.1	2.9	NA	
Arora et al. (2018)	Retrospective	394	99	61	61	27.4	29.0	3.4	2.9	NA	
Laviana et al. (2018)	Retrospective	78	78	NSD		NSD		NSD		NSD	
Dell'Oglio et al. (2019)	Retrospective	384	384	NSD		NSD		NSD		NSD	
Mittakanti et al. (2019)	Retrospective	166	166	60	60	30.3	29.7	3.3	3.1	5.6	5.7
Paulucci et al. (2019)	Retrospective	357	162	59	61	29.7	28.6	3.0	2.9	7	7
Song et al. (2019)	Retrospective	118	89	51.8	53.3	24.1	23.7	3.6	3.5	5.3	5.2
Tai et al. (2019)	Retrospective	102	121	57.5	59.8	22.6	23.2	4.3	3.9	7.9	8.4
Abaza et al. (2019)	Retrospective	107	30	56.3	54.1	32.7	30.6	3.5	3	7.2	7.2
Choi et al. (2020)	Retrospective	310	213	51	50	25.2	25.0	2.9	2.8	7	7
Harke et al. (2020)	Retrospective	176	176	62	63	27	27	NA		NA	
Kobari et al. (2020)	Retrospective	56	65	60	59	25	24	29	27	NA	
Takagi et al. (2020)	Retrospective	48	48	55	55	25	24	3.1	3.0	NA	

Abbreviations: NA, data not available; NSD, no significant difference between the two groups; RP, retroperitoneal; TP, transperitoneal.

**TABLE 2:** Quality assessment of included studies

Study	Selection				Comparability		Exposure			Total points
	REC	SNEC	AE	DO	SC	AF	AO	FU	AFU	
Hughes‐Hallett et al. (2013)	1	1	1	1	1		1		1	7
Tanaka et al. (2013)	1	1	1	1	1		1		1	7
Gin et al. (2014)	1	1	1	1	1		1		1	7
Choo et al. (2014)	1	1	1	1	1	1	1		1	8
Kim et al. (2015)	1	1	1	1	1		1		1	7
Tang et al. (2015)	1	1	1	1	1		1		1	7
Xia et al. (2016)	1	1	1	1	1		1		1	7
Maurice et al. (2017)	1	1	1	1	1	1	1		1	8
Stroup et al. (2017)	1	1	1	1	1		1		1	7
Arora et al. (2018)	1	1	1	1	1	1	1		1	8
Laviana et al. (2018)	1	1	1	1	1		1		1	7
Dell'Oglio et al. (2019)	1	1	1	1	1		1		1	7
Mittakanti et al. (2019)	1	1	1	1	1		1		1	7
Paulucci et al. (2019)	1	1	1	1	1		1		1	7
Song et al. (2019)	1	1	1	1	1	1	1		1	8
Tai et al. (2019)	1	1	1	1	1		1		1	7
Abaza et al. (2019)	1		1	1	1		1		1	6
Choi et al. (2020)	1	1	1	1	1		1		1	7
Harke et al. (2020)	1	1	1	1	1		1		1	7
Kobari et al. (2020)	1	1	1	1	1		1		1	7
Takagi et al. (2020)	1	1	1	1	1		1		1	7

Abbreviations: AE, ascertainment of exposure; AF, study controls for other important factors; AFU, adequacy of follow‐up of cohort (≥80%); AO, assessment of outcome; DO, demonstration that outcome of interest was not present at start of study; FU, follow‐up long enough for outcomes to occur (“long enough” is defined as 1 year); REC, representativeness of the cohort; SC, study control most important factors; SNEC, selection of the non‐posed cohort.

### 3.2 Operating time

**FIGURE 2:**
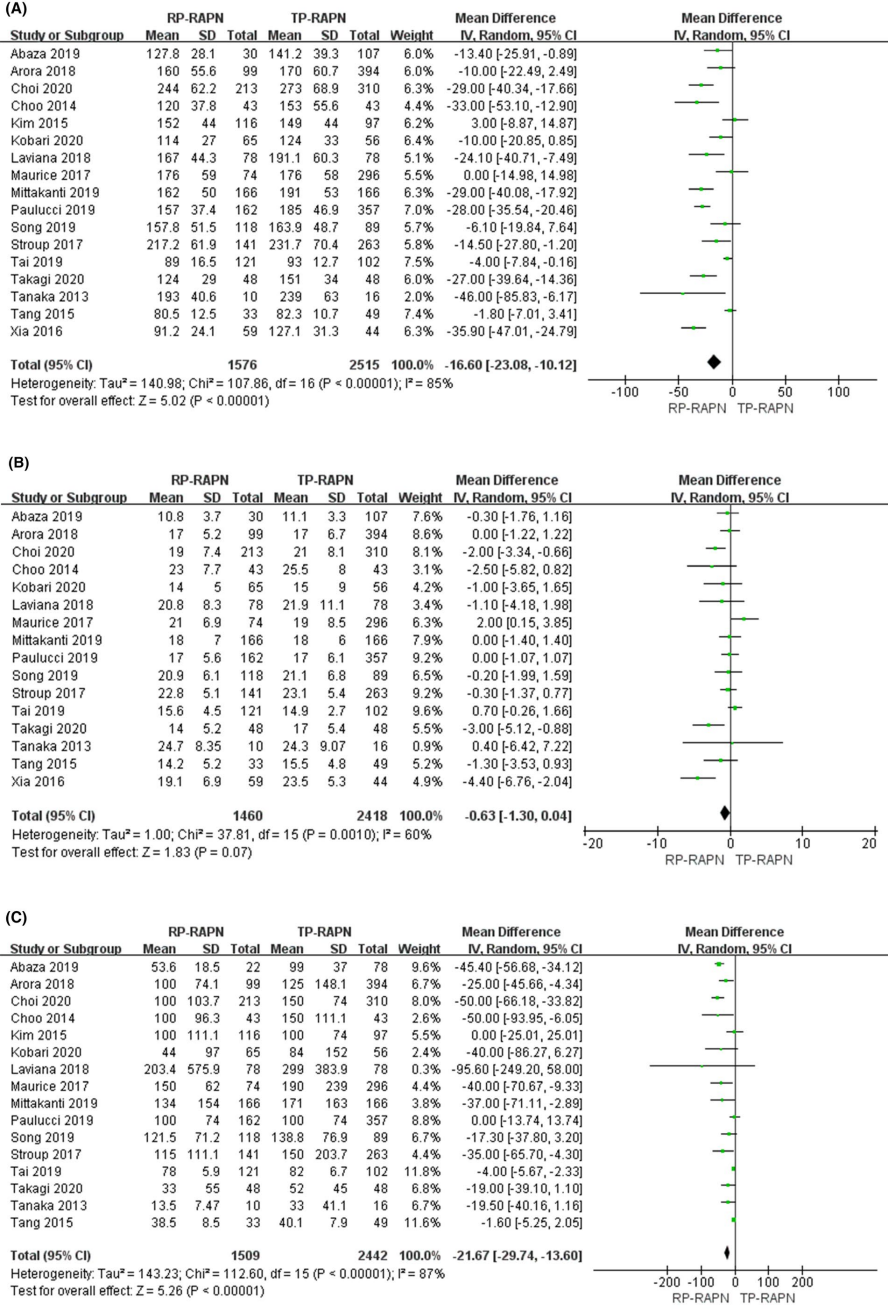
Forest plots of perioperative outcomes: (A) operating time, (B) warm ischemia time, and (C) estimated blood loss

OT data were extracted from 17 studies,^4^, ^23^, ^24^, ^25^, ^26^, ^27^, ^28^, ^29^ totaling 4091 patients (1576 RP‐RAPN vs. 2515 TP‐RAPN). The pooled analysis suggested significant differences for OT between RP‐RAPN and TP‐RAPN (FE model: WMD −16.60; 95% CI −23.08, −10.12; *p* < 0.01; *I* ^2^ = 85%), albeit at a greater heterogeneity (Figure 2A).

### 3.3 Warm ischemia time

Sixteen articles with 3878 patients^4^, ^23^, ^24^, ^25^, ^26^, ^27^, ^28^, ^29^ were analyzed in the study (1460 RP‐RAPN vs. 2418 TP‐RAPN). A random model was used for analysis because there was a high degree of heterogeneity (*I* ^2^ = 60%). There was no statistical difference in WIT across the two approaches (WMD −0.63; 95% CI −1.30, 0.04; *p* < 0.05) (Figure 2B).

### 3.4 Estimated blood loss

Sixteen articles^4^, ^23^, ^24^, ^25^, ^26^, ^27^, ^28^ were included in our study, including 3951 patients: 1509 underwent RP‐RAPN, and 2442 underwent TP‐RAPN. For there is high heterogeneity existed, an RE model was used (*I* ^2^ = 74%). The pooled outcome suggested that EBL in the RP‐RAPN group was similar to that in the TP‐RAPN group (WMD −21.67; 95% CI −29.74, −13.60; *p* < 0.05) (Figure 2C).

### 3.5 Postoperative length of hospital stay

**FIGURE 3:**
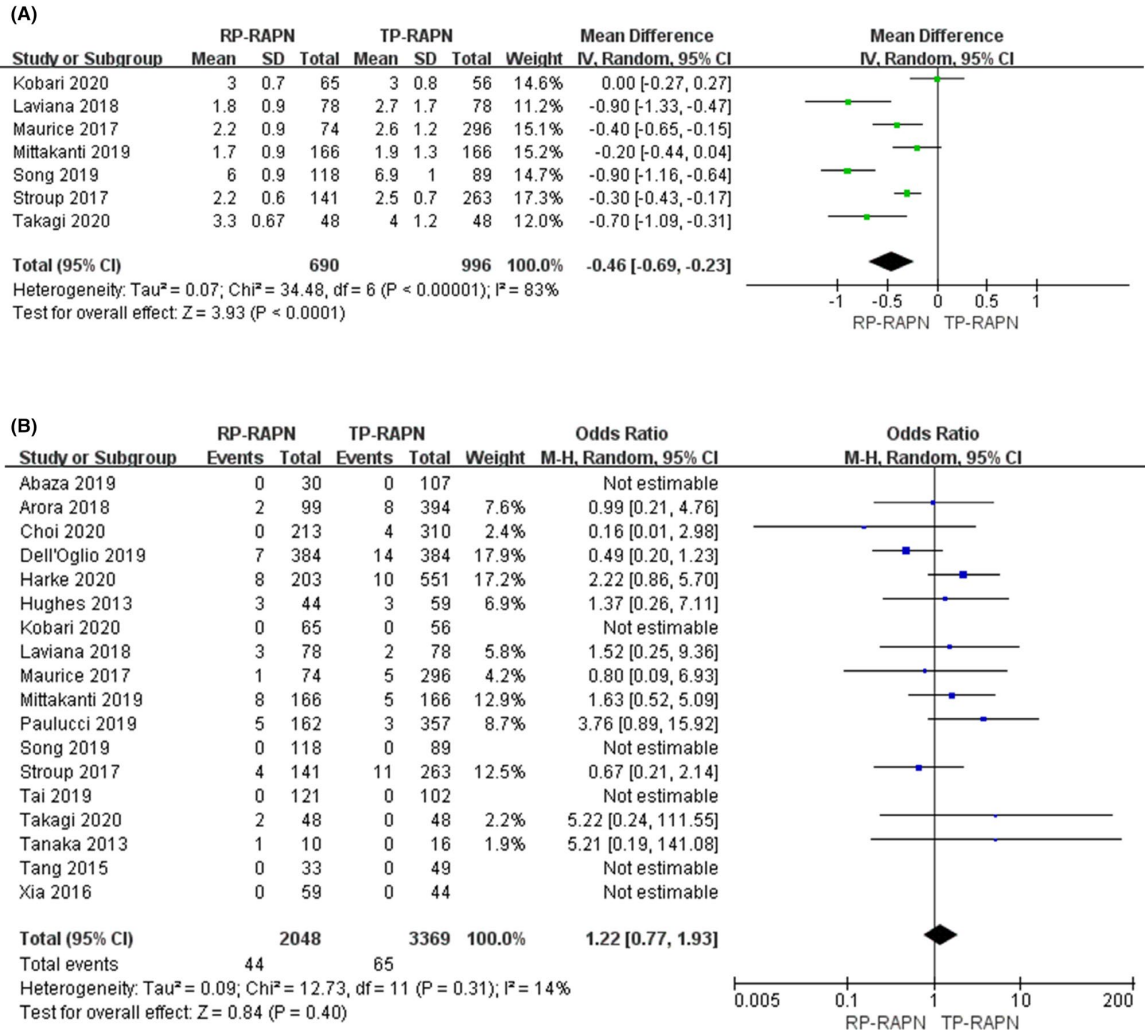
Forest plots of perioperative outcomes: (A) length of postoperative stay and (B) positive surgical margin

PLOS data were extracted from seven studies.^14^, ^15^, ^16^, ^17^, ^23^, ^24^, ^26^ There are 1686 patients in total with 690 RP‐RAPN patients and 996 TP‐RAPN patients. A random model was applied in analysis since there was a high degree of heterogeneity (*I* ^2^ = 83%). There was statistically significant difference in PLOS between the two approaches (WMD −0.46; 95% CI −0.69, −0.23; *p* < 0.05) (Figure 3A).

### 3.6 Positive surgical margin

There was a total of 5777 patients (2048 RP‐RAPN vs. 3369 TP‐RAPN) analyzed in 18 studies.^4^, ^23^, ^24^, ^25^, ^26^, ^27^, ^28^, ^29^, ^30^ Our study demonstrated that there was no significant difference between RP‐RARN and TP‐RAPN (FE model: odds ratio [OR] 1.22; 95% CI 0.77, 1.93; *p* = 0.40; *I* ^2^ = 14%) (Figure 3B).

### 3.7 Major complication

**FIGURE 4:**
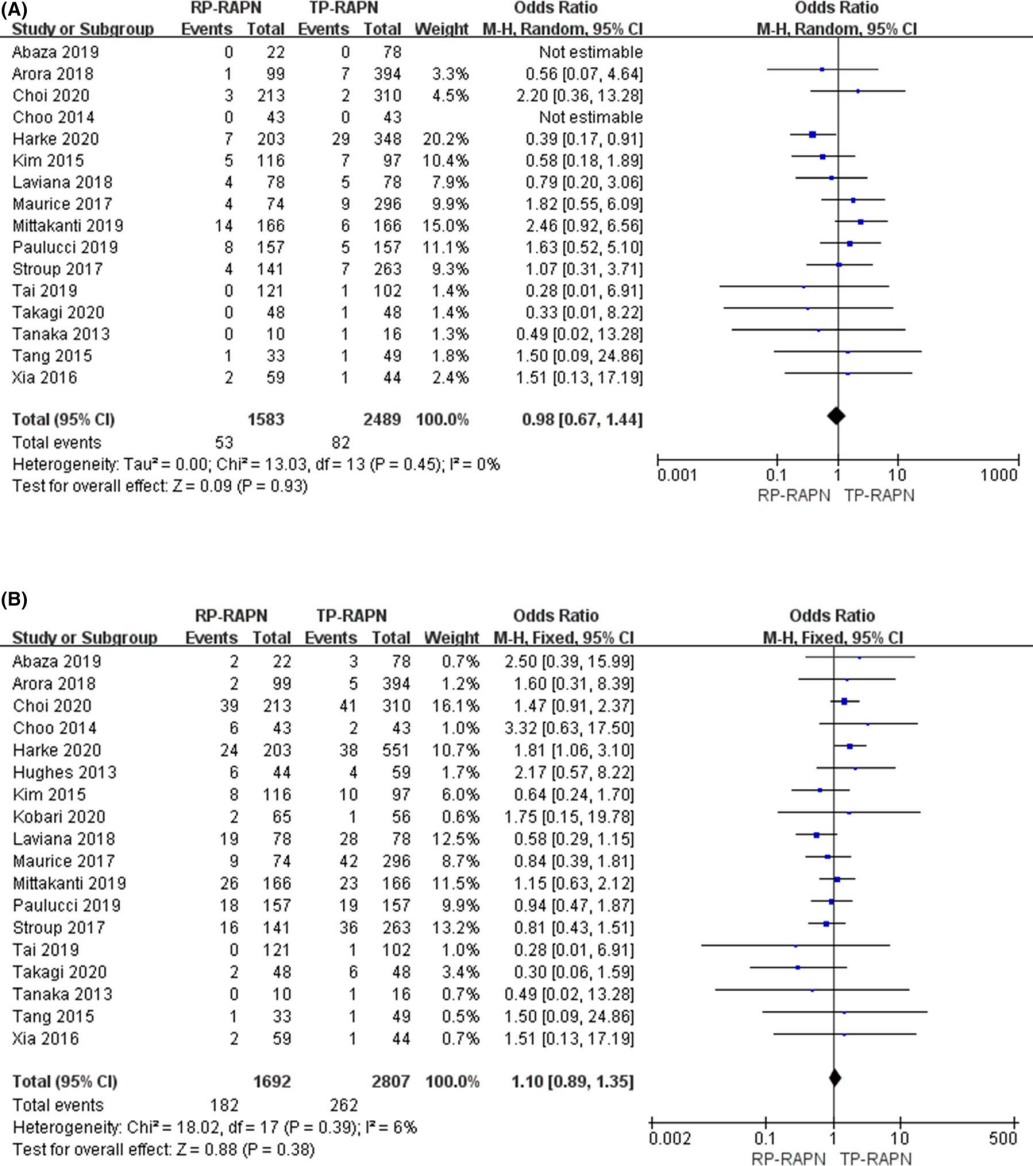
plots of perioperative outcomes: (A) major complication rate and (B) overall complication rate

Sixteen studies were included with a total number of 4072 patients (1583 RP‐RAPN vs. 2489 TP‐RAPN). A fixed model was used for analysis as there was a low degree of heterogeneity (*I* ^2^ = 0%). There was no statistically significant difference in ≥Clavien 3a complication rates across the two approaches (FE model: OR 0.98; 95% CI 0.67, 1.44; *p* = 0.45) (Figure 4A).

### 3.8 Overall complication

Overall complication rate data were extracted from 18 studies.^13^, ^14^, ^15^, ^16^, ^17^, ^24^, ^25^, ^26^, ^27^ There are 4499 patients in total with 1692 RP‐RAPN patients and 2807 TP‐RAPN patients. A fixed model was used for analysis as there was a low degree of heterogeneity (*I* ^2^ = 6%). There were no significant differences between the two groups regarding overall complication rate (FE model: OR 1.10; 95% CI 0.89, 1.35; *p* = 0.38) (Figure 4B).

## 4 DISCUSSION

Minimally invasive PN has traditionally been performed with TP‐RAPN. However, in recent years, RP‐RAPN has been emerged with the advantages of avoiding violation to abdominal organs and so on. The advantages of these two surgical approaches are debatable. From 2019 and 2020, there have been several new high‐quality clinical studies comparing these two surgical approaches in different countries all over the world, which in one hand suggested that many questions are still controversial and confirmed the importance of our meta‐analysis in the other hand. To the best of our knowledge, this is the most up‐to‐date and comprehensive meta‐analysis of reported comparative outcomes of RP‐RAPN versus TP‐RAPN. In the present meta‐analysis, our results showed that RP‐RAPN has non‐inferior and comparable outcomes with TP‐RAPN in WIT, PSM, overall complication rate, and major complication rate. Nevertheless, the findings also indicate that in terms of OT, PLOS, and EBL, RP‐RAPN appears to offer significant superiority over TP‐RAPN. Both transperitoneal and retroperitoneal approaches were applicable in RAPN; however, no recommendations were provided in current urological guidelines nowadays, which makes the decision on the surgical approach difficult.^1^

In our meta‐analysis, OT in RP‐RAPN was significantly shorter than that in TP‐RAPN. It is widely acknowledged that TP approach has a large operative space and there is no need to protect the integrity of the peritoneum, which may significantly shorten the OT, the extreme flexibility of robot‐assisted laparoscopic surgery system offset the technical challenges caused by the narrow working space. According to Ge et al.,^31^ TP‐RAPN was more likely applied in patients with complex and larger tumors due to the larger working space and obvious anatomy structure, which makes it easier to deal with the renal hilum and tumor. Besides, RP‐RAPN can reach the renal hilum more easily, which means less manipulation and shorter OT. All these advantages of RP‐RAPN may explain our finding that the EBL in RP‐RAPN is significantly lower compared to TP‐RAPN in our study with a WMD of 21 ml.

The PLOS in RP‐RAPN was also significantly shorter compared to TP‐RAPN in our meta‐analysis. Shorter length of PLOS was correlated to earlier return of oral intake and bowel function and less postoperative complications. Without the interruption of peritoneum and abdominal organs, RP‐RAPN has a better protection of the intraperitoneal organs from hematoma and urine leaks, consequently fastening the postoperative recovery of bowel. This is in accordance with our finding that the recovery of bowel movement in RP‐RAPN was significantly shorter compared to TP‐RAPN. However, all the four studies was conducted in China, and this may lead to selection bias.

In our study, no significant statistical differences were found between these two surgical approaches in overall complication rate and major complication rate. Dell'Oglio et al.^30^ found a statistically significant difference in postoperative eGFR, while no difference was found in two groups in 1‐year follow‐up. Takagi et al.^26^ reported no difference in postoperative eGFR change in early or 6 months after surgeries. So there is no evidence supporting that statically difference of eGFR change existed between these two surgical approaches. In a previous study, Ge et al.^31^ considered that meta‐analysis is not suitable for eGFR due to the paucity of studies reporting this outcome. However, we have found several studies including perioperative and follow‐up renal function. In our opinion, we are not able to compare the influence on postoperative eGFR because of the inconsistency in the outcomes and the variation in the length of follow‐up among studies. Herein, we propose that authors should measure renal function in an identical way in the future.

Until January 2021, we have not found any prospective randomized double‐blind clinical trials. Nearly all the studies we included were retrospective. In a previous study, Zhu et al.^32^ included two studies solely concentrating on the posterior tumors,^8^, ^13^ and all the results including OT, EBL, and PLOS were consistent with our study. However, tumor location was not the only factor influenced surgeon's choice. Tumor size and proficiency may influence surgeon's subjective assessment of surgical approaches as well. Seven studies try to counterbalance the selection bias by utilizing propensity score matching, which is widely used nowadays in retrospective studies.^8^, ^9^, ^15^, ^17^, ^18^, ^26^, ^30^ Although Gin et al.^9^ have applied the propensity score matching, we are not able to obtain any available data from their study. Zhu et al.^32^ preferred to include comparative studies with matched design and studies with similar baseline characteristics, which is a great inspiration to us. By subgroup analysis of all the studies using propensity score matching and studies concentrating solely on posterior tumor or lateral tumor, we found no significant difference compared to our presented results (OT [WMD −18.33; 95% CI −28.05, −8.62; *p* < 0.01]; PLOS [WMD −0.51 days; 95% CI −0.77, −0.22; *p* < 0.01]; and EBL [WMD −20.31; 95% CI −34.07, −6.54; *p* < 0.05]).

In conclusion, the study demonstrated that RP‐RAPN was not superior to TP‐RAPN in terms of perioperative outcomes. However, more detailed guidelines aiming on the recommendation of surgical approach based on the characteristics of tumor are crucial. Before establishing clinical recommendations, more high‐quality prospective large‐scale randomized controlled trials with long‐term follow‐up are essential. In the case that urologists lack widely acknowledged guidelines, more recommendations on the selection of surgical approaches according to the characteristic of tumor (radius, location, and renal scores) should be presented in the future.

## CONFLICT OF INTERESTS

All the authors declare that they have no conflict of interests.

## ETHICAL APPROVAL

Our study is a secondary research, and all data presented were from published studies. So, there is no need for ethical approval.

## Data Availability

All datasets generated for this study are included in the article/supporting information.

## References

[1] Ljungberg B , Bensalah K , Canfield S , et al. EAU guidelines on renal cell carcinoma: 2014 update. Eur Urol. 2015;67(5):913‐924. 25616710 10.1016/j.eururo.2015.01.005

[2] Campbell S , Uzzo RG , Allaf ME , et al. Renal mass and localized renal cancer: AUA guideline. J Urol. 2017;198(3):520‐529. 28479239 10.1016/j.juro.2017.04.100

[3] Ghani KR , Sukumar S , Sammon JD , Rogers CG , Trinh Q‐D , Menon M . Practice patterns and outcomes of open and minimally invasive partial nephrectomy since the introduction of robotic partial nephrectomy: results from the nationwide inpatient sample. J Urol. 2014;191(4):907‐912. 24184365 10.1016/j.juro.2013.10.099

[4] Abaza R , Gerhard RS , Martinez O . Feasibility of adopting retroperitoneal robotic partial nephrectomy after extensive transperitoneal experience. World J Urol. 2020;38(5):1087‐1092. 31515605 10.1007/s00345-019-02935-z

[5] Arora S , Heulitt G , Menon M , et al. Retroperitoneal vs transperitoneal robot‐assisted partial nephrectomy: comparison in a multi‐institutional setting. Urology. 2018;120:131‐137. 30053396 10.1016/j.urology.2018.06.026

[6] Brozek JL , Akl EA , Jaeschke R , et al. Grading quality of evidence and strength of recommendations in clinical practice guidelines: Part 2 of 3. The GRADE approach to grading quality of evidence about diagnostic tests and strategies. Allergy. 2009;64(8):1109‐1116. 19489757 10.1111/j.1398-9995.2009.02083.x

[7] Choi CI , Kang M , Sung HH , et al. Comparison by pentafecta criteria of transperitoneal and retroperitoneal robotic partial nephrectomy for large renal tumors. J Endourol. 2020;34(2):175‐183. 31621405 10.1089/end.2019.0410

[8] Choo SH , Lee SY , Sung HH , et al. Transperitoneal versus retroperitoneal robotic partial nephrectomy: matched‐pair comparisons by nephrometry scores. World J Urol. 2014;32(6):1523‐1529. 24817141 10.1007/s00345-014-1312-7

[9] Gin GE , Maschino AC , Spaliviero M , Vertosick EA , Bernstein ML , Coleman JA . Comparison of perioperative outcomes of retroperitoneal and transperitoneal minimally invasive partial nephrectomy after adjusting for tumor complexity. Urology. 2014;84(6):1355‐1360. 25288573 10.1016/j.urology.2014.07.045

[10] Harke NN , Darr C , Radtke JP , et al. Retroperitoneal versus transperitoneal robotic partial nephrectomy: a multicenter matched‐pair analysis. Eur Urol Focus. 2020. 10.1016/j.euf.2020.08.012 32912841

[11] Hozo SP , Djulbegovic B , Hozo I . Estimating the mean and variance from the median, range, and the size of a sample. BMC Med Res Methodol. 2005;5:13. 15840177 10.1186/1471-2288-5-13 PMC1097734

[12] Hughes‐Hallett A , Patki P , Patel N , Barber NJ , Sullivan M , Thilagarajah R . Robot‐assisted partial nephrectomy: a comparison of the transperitoneal and retroperitoneal approaches. J Endourol. 2013;27(7):869‐874. 23461381 10.1089/end.2013.0023

[13] Kim EH , Larson JA , Potretzke AM , Hulsey NK , Bhayani SB , Figenshau RS . Retroperitoneal robot‐assisted partial nephrectomy for posterior renal masses is associated with earlier hospital discharge: a single‐institution retrospective comparison. J Endourol. 2015;29(10):1137‐1142. 25816694 10.1089/end.2015.0076

[14] Kobari Y , Takagi T , Yoshida K , Ishida H , Tanabe K . Comparison of postoperative recovery after robot‐assisted partial nephrectomy of T1 renal tumors through retroperitoneal or transperitoneal approach: A Japanese single institutional analysis. Int J Urol. 2020. 10.1111/iju.14424 33145892

[15] Laviana AA , Tan H‐J , Hu JC , Weizer AZ , Chang SS , Barocas DA . Retroperitoneal versus transperitoneal robotic‐assisted laparoscopic partial nephrectomy: a matched‐pair, bicenter analysis with cost comparison using time‐driven activity‐based costing. Curr Opin Urol. 2018;28(2):108‐114. 29278580 10.1097/MOU.0000000000000483

[16] Maurice MJ , Kaouk JH , Ramirez D , et al. Robotic partial nephrectomy for posterior tumors through a retroperitoneal approach offers decreased length of stay compared with the transperitoneal approach: a propensity‐matched analysis. J Endourol. 2017;31(2):158‐162. 27927035 10.1089/end.2016.0603

[17] Mittakanti HR , et al. Transperitoneal vs. retroperitoneal robotic partial nephrectomy: a matched‐paired analysis. World J Urol. 2020;38(5):1093‐1099. 31420695 10.1007/s00345-019-02903-7

[18] Paulucci DJ , Beksac AT , Porter J , et al. A Multi‐Institutional Propensity Score Matched Comparison of Transperitoneal and Retroperitoneal Partial Nephrectomy for cT1 Posterior Tumors. J Laparoendosc Adv Surg Tech A. 2019;29(1):29‐34. 30106606 10.1089/lap.2018.0313

[19] Sterne JA , Hernán MA , Reeves BC , et al. ROBINS‐I: a tool for assessing risk of bias in non‐randomised studies of interventions. BMJ. 2016;355:i4919. 27733354 10.1136/bmj.i4919 PMC5062054

[20] Stang A . Critical evaluation of the Newcastle‐Ottawa scale for the assessment of the quality of nonrandomized studies in meta‐analyses. Eur J Epidemiol. 2010;25(9):603‐605. 20652370 10.1007/s10654-010-9491-z

[21] Cumpston M , Li T , Page MJ , et al. Updated guidance for trusted systematic reviews: a new edition of the Cochrane Handbook for Systematic Reviews of Interventions. Cochrane Database of Systematic Reviews. 2019. 10.1002/14651858.ED000142 PMC10284251 31643080

[22] Higgins JP , Thompson SG . Quantifying heterogeneity in a meta‐analysis. Stat Med. 2002;21(11):1539‐1558. 12111919 10.1002/sim.1186

[23] Song S , He P , Cheng Z . Comparison between robot‐assisted transperitoneal laparoscopic partial nephrectomy and robot‐assisted retroperitoneal partial nephrectomy. Journal of Clinical Urology. 2019;2019(34):845‐849.

[24] Stroup SP , Hamilton ZA , Marshall MT , et al. Comparison of retroperitoneal and transperitoneal robotic partial nephrectomy for Pentafecta perioperative and renal functional outcomes. World J Urol. 2017;35(11):1721‐1728. 28656359 10.1007/s00345-017-2062-0

[25] Tai S , Zhou J , Shi H , et al. Analysis of therapeutic effect among different accesses robot‐assisted laparoscopic partial nephrectomy. Journal of Clinical Urology. 2019;19(34):18‐21.

[26] Takagi T , Yoshida K , Kondo T , et al. Comparisons of surgical outcomes between transperitoneal and retroperitoneal approaches in robot‐assisted laparoscopic partial nephrectomy for lateral renal tumors: a propensity score‐matched comparative analysis. J Robot Surg. 2021;15(1):99‐104. 32358741 10.1007/s11701-020-01086-3

[27] Tanaka K , Shigemura K , Furukawa J , et al. Comparison of the transperitoneal and retroperitoneal approach in robot‐assisted partial nephrectomy in an initial case series in Japan. J Endourol. 2013;27(11):1384‐1388. 24044768 10.1089/end.2012.0641

[28] Tang H , Zhang Z , Zhou W , et al. Comparison of the transperitoneal and retroperitoneal approach in robotic‐assisted laparoscopic partial nephrectomy in treatment of renal carcinoma. Chinese Clinical Oncology. 2015;2015:1128‐1131.

[29] Xia D , Wang P , Qin J , et al. Comparison of transperitoneal and retroperitoneal approach in robotic‐assisted laparoscopic partial nephrectomy. Chinese Journal of Urology. 2016;37(2):81‐84.

[30] Dell'Oglio P , Naeyer GD , Xiangjun L , et al. The Impact of surgical strategy in robot‐assisted partial nephrectomy: is it beneficial to treat anterior tumours with transperitoneal access and posterior tumours with retroperitoneal access? Eur Urol Oncol. 2021;4(1):112–116. 31411997 10.1016/j.euo.2018.12.010

[31] Ge S , Chen L , Tai S . Comparison of therapeutic effects among different surgical approaches in robot‐assisted partial nephrectomy: a systematic review and Meta‐analysis. J Endourol. 2020. 10.1089/end.2020.0432 33076702

[32] Zhu D , Shao X , Guo G , et al. Comparison of outcomes between transperitoneal and retroperitoneal robotic partial nephrectomy: a meta‐analysis based on comparative studies. Frontiers in Oncology. 2021;10:592193. 33489891 10.3389/fonc.2020.592193 PMC7819878

